# Preparedness of emergency departments in northwest England for managing chemical incidents: a structured interview survey

**DOI:** 10.1186/1471-227X-7-20

**Published:** 2007-12-20

**Authors:** Jane Williams, Darren Walter, Kirsty Challen

**Affiliations:** 1 Emergency Department, University Hospital of South Manchester, Manchester, UK

## Abstract

**Background:**

A number of significant chemical incidents occur in the UK each year and may require Emergency Departments (EDs) to receive and manage contaminated casualties. Previously UK EDs have been found to be under-prepared for this, but since October 2005 acute hospital Trusts have had a statutory responsibility to maintain decontamination capacity. We aimed to evaluate the level of preparedness of Emergency Departments in North West England for managing chemical incidents.

**Methods:**

A face-to-face semi-structured interview was carried out with the Nurse Manager or a nominated deputy in all 18 Emergency Departments in the Region.

**Results:**

16/18 departments had a written chemical incident plan but only 7 had the plan available at interview. All had a designated decontamination area but only 11 felt that they were adequately equipped. 12/18 had a current training programme for chemical incident management and 3 had no staff trained in decontamination. 13/18 could contain contaminated water from casualty decontamination and 6 could provide shelter for casualties before decontamination.

**Conclusion:**

We have identified major inconsistencies in the preparedness of North West Emergency Departments for managing chemical incidents. Nationally recognized standards on incident planning, facilities, equipment and procedures need to be agreed and implemented with adequate resources. Issues of environmental safety and patient dignity and comfort should also be addressed.

## Background

Exposure to hazardous chemicals can occur as a result of a wide range of events including through accidental release, industrial accident and by act of terrorism. Around 1300 chemical incidents occur in the UK each year, most involving fewer than 10 casualties [1]. Most published UK guidance on the management of major chemical incidents assumes or dictates that casualties will be decontaminated at the scene of the incident [2] and then transported to hospital. This pattern of patient behaviour was not substantiated by experience in Japan, where 85% of patients following the Tokyo subway sarin attacks in March 1995 self-transported to hospital [3], or in the United States, where between 1995 and 2001 15 Emergency Department (ED) personnel were injured as a result of contaminated casualties in 6 different incidents with agents including hydrofluoric acid, acetone, hydrochloric acid and chlorine [4]. There are also case reports where contamination of an ED by a patient has resulted in the department having to be closed for a period [5].

Serious inadequacies have been highlighted in the preparedness of UK Emergency Departments for the management of chemical incidents. In 2000, 76% of EDs did not have satisfactory premises for decontamination and 66% lacked protective equipment for staff [6], whilst 23% of those departments with 20,000 or more new attendances per year had no capacity to decontaminate patients or staff [7]. It might have been hoped that the events of September 11^th ^2001 and the subsequent raising of awareness of chemical threats might have improved the situation. This has not however been substantiated by the National Audit Office [8] or a more recent study which found that only 82% of UK EDs had trained staff in the use of Personal Protective Equipment (PPE). The same study identified a number of problems with the PPE currently in use, notably leaks around the foot area and problems with sizing [9].

With the implementation of the Civil Contingencies Act in October 2005, NHS Acute Trust Chief Executives acquired statutory responsibility for the adequacy of their facility's plans for dealing with major incidents, including the management of chemically contaminated casualties. The Department of Health guidance "Beyond a Major Incident" states:

"It is likely that the current equipment, preparations and general capability for decontamination of small numbers of casualties at most hospitals would be put under severe strain by the scale and circumstances of mass casualty incidents with contamination. The hospital must address this by developing a mass casualty plan, in close collaboration with police and fire services for control of the site, mass decontamination and to deal with self referrals. The special arrangements necessary will include crowd management and a triage/assessment facility as an adjunct to the A&E Department to avoid cross contamination [10]."

We therefore aimed to assess the preparedness of hospitals in our region for the management of a chemical incident.

## Methods

A 34-item questionnaire was constructed by one of the authors (JW) to address decontamination incident planning, staff training, estate facilities, equipment, protocols, water supply for patient decontamination and issues of dignity, privacy and comfort (see Additional file 1). Each of the 18 EDs in the North West England region was contacted and the questionnaire was administered in person by JW at interviews with either the nurse manager or another member of staff with expertise in decontamination, nominated by the nurse manager. The interviewee was pre-informed of the topics to be covered in the interview by fax. Any questions which could not be answered by the interviewee were addressed by a telephone call to the Estates Department of the hospital in question. Anonymised data was entered into SPSS 10.1.4 (SPSS inc^®^) for analysis.

## Results

All 18 Emergency Departments in the region participated. In 6 the nurse manager was interviewed personally, whilst, in the remaining 12, another person was nominated (10 other senior nurses, 1 consultant, 1 emergency planner).

### Planning and training

16 of the 18 departments had a formal written incident plan for the management of chemical incidents, but only 7 of these had it easily accessible at the time of interview. 12 respondents were confident in their ability to wash down a patient, but only 5 were sure of the time required for decontamination. 16 interviewees felt they knew which agencies to contact in the event of a chemical incident, of which 9 had the appropriate telephone numbers immediately to hand. Agencies which would be contacted were:

• Emergency services (fire, police, ambulance): 18

• Environment Agency: 17

• Health Protection Agency (Chemical Hazards & Poisons): 17

• Local authority: 14

• Water company: 14

Training relating to response to a chemical incident was ongoing in 12 departments. Of the remaining 6, 3 could not quantify the training levels of current staff, 2 relied on managers and senior doctors being trained and 1 had "some" senior staff trained. Of the 12 departments with ongoing training, this was mixed theory and practice in 11 and practice assembly of the decontamination unit in 1. Sessions were repeated more than twice a year in 3 departments, between once and twice a year in 3, less than once a year in 2, and were considered to be a one-off in 4.

### Facilities and equipment

All departments had a designated decontamination area. In 17 this was outside and of these 16 used PLYSU^® ^units and 1 an Airshelta^® ^unit. It was unknown in the department with an indoor decontamination facility whether its ventilation could be separated from that of the rest of the hospital. 11 interviewees felt that their departments had adequate Personal Protective Equipment (PPE) for staff involved in decontamination.

### Water supply and disposal

All departments were using water from the hospital mains, 17 directly to supply the showerhead and 1 to fill buckets. All 17 using a showerhead confirmed that it had no ability to reach contaminated water. 8 departments had a double check valve fitted to the water supply to ensure no backflow into the hospital mains was possible, 1 department knew that there was no safety valve and 9 were unsure of safety precautions. The water supply for decontamination was run weekly in 5 departments, never in 2 and at unknown intervals in the remaining 11.

7 departments used standard mains cold water for decontamination, 5 because work to heat the water had yet to be carried out and 2 for unknown reasons. 11 departments used a hot/cold water mix of above 38°C, all for reasons of patient comfort. Water temperature was thermostatically controllable in only 3 of the 17 departments using showers.

In 11 departments, contaminated water could only be contained up to 500 litres (under 1 hours' worth of decontamination). 4 departments could contain up to 1000 litres (between 1 and 2 hours' worth) of effluent and 3 could contain more. Once containment capacity had been exceeded, 4 departments would allow overflow into drains, 3 could bypass to further storage tanks, 7 would stop decontaminating and 4 were unclear of their further actions. 2 departments had formal contracts with private waste disposal companies for the disposal of contaminated water, 11 were relying on the fire service and 5 planned to seek advice from the Health Protection Agency or Environment Agency. In the event of disposal into the drain, 2 departments could easily differentiate between surface and foul water drains, 1 had a combined drain system, 1 only drained to an underground tank (from an indoor decontamination facility) and the remaining 14 could not identify surface and foul water disposal facilities but felt that the hospital Estates Department would be able to do so. When discharging into mains drainage, 5 departments planned to add fluorescein dye to the contaminated water to aid identification at downstream sewage processing facilities.

### Patient privacy, dignity and comfort

6 departments provided a sheltered area for patients to remove contaminated clothing before decontamination; this was the ambulance bay canopy in 3, in a specific separate unit in 1 and in the decontamination unit itself in 2. Temporary clothing consisted of Rotecno^® ^purpose-bought clothing in 2 departments, paper clothes in 9, hospital gowns and blankets in 4, hospital gowns only in 2 and hospital blankets only in 1.

9 departments felt they could adequately maintain patient dignity during decontamination, 7 using hospital screens, 1 a specific unit and 1 the decontamination unit itself. 1 department planned to separate men and women using hospital screens.

## Discussion

### Planning and training

It is alarming that 2 departments lacked a written plan for chemical incidents and a further 9 could not access theirs at the time of interview. This suggests that the majority of departments would not have a plan available in the event of an incident, which is likely to result in delays or even failures in contacting appropriate personnel from within and outside the hospital and in substandard handling of patients, possibly with unnecessary risks to staff. A recent Delphi study into chemical incident management suggested training to a national standard for all ED medical and nursing staff [11]. It is apparent that this is not the current standard, with two-fifths of departments having no current training programme and one-fifth being unable to identify trained staff in the department.

Chemical incidents require the use of specialist and complex equipment (both PPE and decontamination tents) and, given our findings, it is unlikely that these could be efficiently used. It is of equal concern that only half of departments had telephone contact numbers for the other agencies likely to be involved in a chemical incident response immediately to hand. Although this might not immediately compromise patient care, delay in contacting agencies such as the Health Protection Agency or the Environment Agency could impact adversely and unnecessarily on the safety of the local area and the environment in general.

### Facilities and equipment

Although it is reassuring to find that all departments surveyed have a designated area for decontamination, several interviewees expressed concerns over the speed with which external shelters could be erected; it is recommended that units should be erected and ready for use within 15 minutes [11]. Several departments expected it to take around 45 minutes to have a functioning unit.

Although all decontamination units had a 'dirty' entrance and 'clean' exit demarcated, one third of the departments had yet to consider how to prevent casualties cross-contaminating themselves and each other. It is particularly worrying that one-fifth of departments felt they lacked the equipment necessary for safe decontamination and a further fifth had not examined the equipment closely enough to identify missing or problematic equipment.

### Water supply and disposal

It is encouraging that 17 of 18 departments are using a shower facility to decontaminate rather than the bucket system which compromises the ability of staff to rinse off contaminant and detergent and which provides less dilution of the contaminant. The use of mains cold water in two-fifths of departments, however, compromises patient compliance and comfort and increases the risk of hypothermia, especially in vulnerable groups such as the elderly and young children. Although there is little published evidence on the optimal water temperature for decontamination (to balance increased efficiency of cleaning against peripheral vasodilation and increased transcutaneous absorption of contaminant at higher temperatures), most experts contacted in the course of this survey suggested a temperature midway between cold and body temperature. No department surveyed was currently using this, and only 3 had thermostatically controlled showers which would make it possible.

Over half of the departments surveyed could not confirm the presence of any backflow protection on their water source from the hospital mains, risking leak of any contaminants into the main hospital supply (and in contravention of the Water Fittings Regulations 2000 – personal communication, Water Regulations Advisory Scheme). It is also apparent that patients and staff may be put at risk from Legionella during decontamination; three-quarters of departments were unable to confirm the regular running of decontamination water points [12].

The appropriate management of contaminated effluent water is still under debate and it is still argued in some quarters that dilution of contaminant with large quantities of water renders it acceptable for discharge into sewerage systems [6]. Specific protocols for the management of contaminated water at hospital sites are not available, but Water UK (representing the utility companies) states as a general principle that "contaminants and contaminated materials should be contained either at the scene or in a holding tank until they have been properly identified". Their protocol for medium scale to mass decontamination in essence comprises a hierarchy of containment (with the expectation that the Fire Service will have 1 hour's containment capacity), discharge to foul sewer and finally discharge to surface water drains [13]. Although three-quarters of departments would attempt to contain contaminated water, three-fifths had capacity of less than one hour. Were this to be exceeded and contaminated water directed into the sewerage system, 14 departments could not independently identify foul and surface water drains, and only one-quarter would add fluorescein dye to the effluent to facilitate its identification in the water system for downstream handling. We would advocate increased contact between hospital emergency planners and utility providers to mitigate this.

### Patient privacy, dignity and comfort

Our findings suggest that little consideration has been given to issues of patient compliance, dignity, comfort and even safety. Two thirds of departments surveyed could provide no shelter for patients disrobing before decontamination, even though the process of decontaminating each patient may take up to 10 minutes and casualties would be expected to remain outside the Emergency Department until their turn. Even after decontamination, two-fifths of departments relied on hospital gowns to clothe their patients, thereby failing to offer full body protection and compromising modesty. Anecdotal reports made during this survey suggest that some patients may refuse treatment rather than compromise their modesty; "one man left the scene because he refused to take his clothes off outside...a crowd was forming around the barriers...".

It is clear that a training gap exists in the management of contaminated casualties in our region. We suggest that this reflects a lack of training capacity both in terms of time and finance, given the multiple constraints affecting most Emergency Departments (notably the 4-hour target, and other mandatory training to fulfill Health and Safety requirements). Questions of who and how many to train, and to what level, remain unclear, and it has been our experience that decontamination management is often sacrificed to competing, more immediately-measurable priorities.

## Conclusion

We have identified major inconsistencies in preparedness for chemical incidents amongst Emergency Departments in North West England, with deficiencies in planning, facilities, equipment and training. Clear national guidelines are still required to address these problems and until standards are set and enforced it is likely that these inconsistencies will remain.

## Competing interests

The author(s) declare that they have no competing interests.

## Authors' contributions

DW conceived the study. DW and JW constructed the questionnaire and JW carried out interviews. All authors contributed to analysis and drafting of the paper and read and approved the final manuscript.

## Pre-publication history

The pre-publication history for this paper can be accessed here:



## Supplementary Material

Additional file 1 Questionnaire Click here for file
