# Supplementary material for: Preparedness of emergency departments in northwest England for managing chemical incidents: a structured interview survey
Source: BMC Emerg Med. 2007 Dec 20;7:20. doi: 10.1186/1471-227X-7-20 (PMC2241633; doi:10.1186/1471-227X-7-20)
Supplement: Additional file 1 — Questionnaire [file 1471-227X-7-20-S1.doc]

**Additional file 1 – Questionnaire**

Please tick the most accurate answer(s)

- Name of hospital……………….
- Position of person(s) filling in questionnaire:
  - Nurse manager
  - Nurse (state grade……………)
  - Emergency Planner
  - Consultant
  - Doctor (state grade……………….)
  - Estates staff
  - Other (specify…………….)
- Please could you supply a contact name and telephone number for any necessary clarification or follow-up enquiries …………………………………………………………………………

GENERAL QUESTIONS

Plans:

- Does your department have a formal written incident plan in place already for decontamination incidents?
- Yes
- No

If yes, do you have a copy of the plan that I can see/take a copy away with me

- Yes
- No (Reason why……………..)

If no, is:

- Plan being developed at present
- Plan thought to be important but not yet formally discussed
- Not considered a priority at moment

Training;

- Do you run a staff training programme for managing decontamination incidents
- Yes - Is this using the NHS structured approach to chemical incidents? Y / N
- No
  - If yes, which members of staff receive this training?
  - all A&E staff
  - only full time A&E staff
  - named / specified persons (specify…………………)
  - others (specify ………………..)
  - How many staff are trained in total? …………..
  - If yes, does the training take place as:
- classroom sessions (theoretical),
- practice scenarios (practical)
- theoretical and practical
- handouts
- not at all
  - If yes, how often is this training carried out:
- Less than every 2 years
- Every 1-2 years
- Every 6 months -1 year
- More than every 6 months

Facilities:

- Does your department have a designated area for decontamination
- Yes
- No

If yes, is the area

- Inside
- In the entrance of the emergency department
- Outside

If inside, is it

- a separate facility eg. Purpose built for decontamination
- in the main A&E dept

If inside, is the ventilation system

- - - - Separate from that of the rest of the hospital
      - Has the ability to be separated from the rest of the hospitals ventilation (how quickly? By who?)
      - The same as the rest of the hospital
      - Don’t know

If outside, would you use?

- - PLYSU inflatable decontamination units
  - Inflatable unit (not PLYSU) – make: …………..
  - Separate building (with own entrance and exit)
  - On the pavement using hoses (no shelter)
  - Other

Equipment:

- What decontamination kit has been provided to you?
- Have you subsequently purchased further kit?
  - What?
  - Why?
- Do you feel that the equipment your department has at present is sufficient?
- Yes
- No
- Don’t know

Protocols / Procedures:

- Do you know the protocol for washing down contaminated patients?
- Yes (Describe…………………………………)
- No
- How long do you decontaminate patients for?
- Yes (Describe………………………………)
- No
- Are you clear about which agencies to contact in the event of a chemical incident?
- Yes
- No
- Which agency or agencies would you contact in the event of a decontamination incident (the reason for contacting them to be written next to each agency)
- EA
- Water Company
- HPA
- Local Authority
- Emergency Services
- Other (specify………………………)
- How accessible are the telephone numbers of the agency or agencies, that you have ticked above:
- Rapidly and easily
- Not immediately but in department
- Not available in department

**WATER**

Water Supply:

- Is the water used for decontamination, coming from:
- Hospital mains water (directly)
- Hospital mains water (with air break)
- Separate storage tank (always filled)
- Separate storage tank (filled when needed)
- Other………………….
- Could you give reasons for the choice of water supply?
  - Yes (Describe…………………………)
  - No
- How often is the water supply for decontamination run?
- Every day
- More than once a week
- More than once a fortnight
- More than once a month
- More than once 6-monthly
- Yearly
- Never
- Don’t know
- How long can the water supply run for:
- <1 hour
- 1-2 hours
- 2-3 hours
- 3-4 hours
- >4 hours
- Don’t know
- Do you know what considerations have been given to prevent backflow of contaminated water into the mains system?
- Yes (Describe………………………………)
- No (Do you know who would……………..)
- Does the shower head in the decontamination facility have the ability to reach the water collecting in the base of the decontamination unit?
- Yes
- No

Water temperature:

- Is the water supply for decontamination
- Cold
- Luke warm <37°C
- Warm >38°C
- Please could you explain why this temp is used?
- If the water is heated how is this done (Describe)
- Is the water thermostatically controllable
- Yes
- No

Water Effluent:

- Can your department contain contaminated water effluent?
- Yes
- No
- If the water is contained, how long can it be contained for
- <1hour
- 1-2 hours
- >2hours
- If the water is contained , what is the form of containment?
- Underground tank
- Pumped into above ground tank
- Succession of small portable tanks
- Decontamination unit sump
- Other (describe………………………….)
- What is the containment volume?……………
- When the container is full, does further water:
- Overflow into the drain thereby flushing out existing contents
- Bypass the tank
- Other
- Who disposes of the contained contaminated water?
- Private company
- Fire service
- Environment agency
- Water company
- HPA
- Other (specify………………)

Is this disposal:

- a contracted arrangement?
- Will emptying be arranged as required?
- What happens to the water if containment no longer possible?
- Discharges to foul drain
- Discharges to storm/surface water drain
- EA/HPA decision
- Don’t know
- Other………………………………
- If foul or storm drains are to be used:
  - Are the different drains identified
- Yes
- No
  - Are the different drains marked for easy identification
- Yes
- No
  - If no to above questions, would you know how to tell them apart
- Yes
- No
  - Would fluorescein dye be added to the contaminated water before discharging to the drains?
- Yes
- No

**Patient comfort, privacy and dignity:**

- Where the patients are expected to remove their clothes is there:
  - Protection from the elements (shelter)
    - Yes (Details……………………………………….)
    - No
  - Ability to maintain dignity
- Yes (Details…………………………………)
- No
- Ability to separate men and women
- Yes (Details……………………………………)
- No
- Once clothes are removed do you have provision for temporary clothing eg. Paper clothes
- Yes (describe……………………………)
- No
- On a scale of 1 (unprepared) to 10 (very prepared) how well prepared you think your A&E department is for a decontamination incident?
- On a scale of 1 to 10 how prepared does the interviewer think this department is?

Name of interviewee Signature of interviewee

Date………………………. Photography approved Y / N
